# ROS‐ATM‐CBP axis‐mediated PARP1 lactylation aggravates doxorubicin‐induced cardiotoxicity

**DOI:** 10.1002/ctm2.70745

**Published:** 2026-08-17

**Authors:** Wenbin Wang, Yingxian Sun

**Affiliations:** ^1^ Department of Cardiology First Hospital of China Medical University Shenyang China

**Keywords:** cardiotoxicity, CBP, doxorubicin, lactylation, PARP1, ROS

## Abstract

Doxorubicin (DOX) is a potent antineoplastic agent, but its clinical application is limited by the life‐threatening cardiotoxic effects. Poly(ADP‐ribose) polymerase 1 (PARP1) transfers ADP‐ribose groups from donor NAD^+^ molecules onto target substrates upon oxidative stress, which may promote apoptosis and myocardial injury. The detailed molecular pathways of PARP1 activation, however, remain unclear. Here, we found that reactive oxygen species (ROS) function as signalling molecules to promote CBP‐mediated PARP1 lactylation in an ATM‐dependent manner, which induces PARP1 PARylation and ultimate cell apoptosis. We identified CBP and SIRT1 as specific lactyltransferase and delactylase of PARP1, respectively. Besides, the lactylation of PARP1 depends on the LDHA activity and lactate production. Moreover, Myocardium‐specific CBP knockout mice decrease PARP1 lactylation and prevent doxorubicin‐induced cardiac dysfunction. Our findings unravelled that excessive oxidative stress‐induced ROS accumulation activates the ATM‐CBP‐PARP1 signalling pathway via a lactylation–PARylation cascade to promote apoptosis and myocardial injury.

## Highlights

- ROS function as signalling molecules to promote CBP‐mediated PARP1 lactylation in an ATM dependent manner, which induces PARP1 PARylation and ultimate cell apoptosis.
- CBP and SIRT1 as specific lactyltransferase and delactylase of PARP1.
- Lactylation of PARP1 depends on the LDHA activity and lactate production.
- Myocardium‐specific CBP knockout mice decrease PARP1 lactylation and prevent doxorubicin‐induced cardiac dysfunction.

## Graphical abstract

1. ROS function as signalling molecules to promote CBP‐mediated PARP1 lactylation in an ATM dependent manner, which induces PARP1 PARylation and ultimate cell apoptosis.
2. CBP and SIRT1 as specific lactyltransferase and delactylase of PARP1.
3. Lactylation of PARP1 depends on the LDHA activity and lactate production.
4. Myocardium‐specific CBP knockout mice decrease PARP1 lactylation and prevent doxorubicin‐induced cardiac dysfunction.

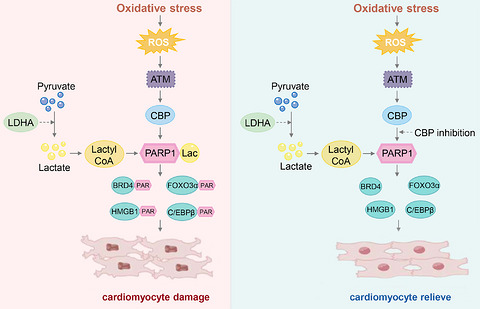

## 1 INTRODUCTION

Poly(ADP‐ribose) polymerase 1 (PARP1) is the first member of the PARP family to be discovered,^1^ the major functions of which depend on its catalytic activity.^2^ As an energy‐consuming enzyme, PARP1 transfers ADP‐ribose groups from donor NAD^+^ molecules onto target protein substrates, including histones, high‐mobility‐group proteins, transcriptional and epigenetic factors, as well as to itself after activation by oxidative stress. Through poly(ADP‐ribosyl)ation (PARylation) of target protein substrates, PARP1 participates in a variety of cellular processes, such as apoptosis and transcriptional regulation.^3^, ^4^, ^5^ However, due to its capacity to deplete cellular energetic pools, excessive intracellular damage generates large branched‐chain PAR polymers, which finally result in cell dysfunction and even cell death.^3^, ^6^, ^7^, ^8^ Therefore, the activation intensity of PARP1 may be a key factor in regulating cell death or survival after oxidative stress. Current studies have confirmed that inhibiting the over‐activation of PARP1 alleviates myocardial injury, as proved by multiple pharmacological methods.^9^, ^10^, ^11^, ^12^, ^13^ Although the functions of PARP1 have been well studied, the molecular mechanism by which PARP1 activation occurs remains unknown.

Post‐translational modifications (PTMs) have been suggested to be critical in regulating the activity, stability, and folding of proteins by inducing their covalent linkage to new functional groups, including phosphate groups, methyl groups, acetyl groups and ubiquitin,^14^, ^15^, ^16^, ^17^ thereby further affecting cellular homeostasis and disease progression. Besides the PARylation modifications to PARP1 itself, other PTMs of PARP1 have also been reported in relation to different diseases. For example, PARP1 is SUMOylated by PIAS4 and subsequently ubiquitylated by the SUMO‐targeted E3 ubiquitin ligase RNF4, which promotes the removal of trapped PARP1 from chromatin in homologous recombination‐defective tumour cells and patient‐derived tumour organoids.^18^ Furthermore, deacetylation‐dependent regulation of PARP1 by SIRT2 was found to dictate ubiquitination of PARP1, which mitigated oxidative stress‐induced vascular injury.^19^ The Src‐mediated the Y992 phosphorylation of PARP1 induced hepatocellular carcinoma (HCC) resistance to PARP1 inhibitors.^20^ The receptor tyrosine kinase c‐Met increases PARP1 enzymatic activity by phosphorylating PARP1 at Tyr907 and reduces binding to a PARP inhibitor, thereby rendering cancer cells resistant to PARP inhibition.^21^ The 2'‐O‐methylation of PARP1 by C/D box small nucleolar RNA SNORD104 increased the stability of PARP1 mRNA and promoted its nuclear localization.^22^ However, whether PARP1 undergoes other important PTM remains unknown.

Lactate was previously thought to be an intermediate metabolite and exerts its metabolic functions in the tricarboxylic acid (TCA) cycle.^23^ Current studies have demonstrated that lactate can also induce a new PTM called lactylation, which alters the function of histone or non‐histone proteins by modifying lysine residues.^24^ It was reported that the lactylation of MRE11 by the CBP lactyltransferase in response to DNA damage promotes its binding to DNA, facilitating DNA end resection and homologous recombination in cancer cells.^25^ The lactylation at the K673 site of fatty acid synthase inhibited its activity, which mediates the downregulation of liver lipid accumulation by mitochondrial pyruvate carrier 1 (MPC1).^26^ The lactyltransferase AARS2 and delactylase SIRT3 regulated mitochondrial protein lactylation (PDHA1 and CPT2), integrating intracellular hypoxia and lactate signals to regulate Oxidative phosphorylation (OXPHOS).^27^ Although the lactylation of PARP1 was mentioned,^28^ the molecular mechanism by which PARP1 lactylation occurs and its functions remain unknown.

Here, we identified that CBP and SIRT1 work as lactyltransferase and delactylase of PARP1, respectively. Moreover, the lactylation of PARP1 highly depends on LDHA activity. Importantly, reactive oxygen species (ROS) are key signalling molecules for activating the ATM‐CBP pathway under oxidative stress, which mediates PARP1 lactylation and subsequent PARylation. Myocardium‐specific CBP knockout mice decrease PARP1 lactylation and prevent doxorubicin‐induced cardiac dysfunction. Our findings demonstrated that oxidative stress‐induced ROS accumulation activates the ATM‐CBP‐PARP1 signalling pathway via a lactylation–PARylation cascade to promote apoptosis and myocardial injury.

## 2 RESULTS

### 2.1 CBP is the lactyltransferase for PARP1 lactylation

**FIGURE 1:**
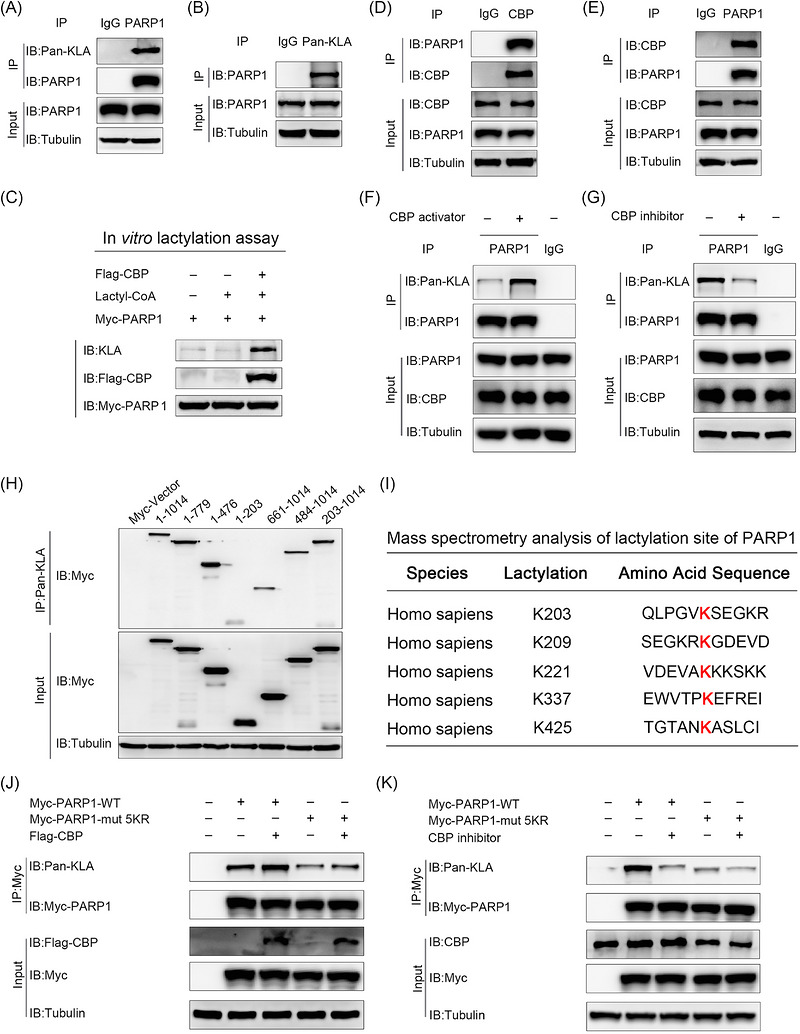
CBP is the lactyltransferase for PARP1 lactylation. (A, B) Lactylation of endogenous PARP1 was determined by Co‐IP using anti‐PARP1 or anti‐PanKla antibodies in HL‐1 cells. (C) CBP can specifically catalyze lactylation of PARP1 in vitro. (D, E) Co‐IP of endogenous proteins from HL‐1 cell lysates using anti‐CBP or Anti‐PARP1 antibody, followed by immunoblot of CBP, PARP1 and Tubulin. (F, G) HL‐1 cells treated with or without CBP activator or CBP inhibitors were lysed and immunoprecipitated using anti‐PARP1 antibody, followed by detection of Pan‐KLA. (H) Full‐length Myc‐PARP1 or six truncated Myc‐PARP1 plasmids were transfected, respectively, and total lysates were subjected to immunoprecipitation with anti‐Myc antibody, followed by detection of Pan‐KLA. (I) The mass spectrometry analysis results of the lactylation site of PARP1. (J) HL‐1 cells transfected with Myc‐PARP1‐WT, Myc‐PARP1‐mut 5KR and Flag‐CBP were lysed and immunoprecipitated using anti‐Myc antibody, followed by detection of Pan‐KLA. (K) HL‐1 cells transfected with Myc‐PARP1‐WT and Myc‐PARP1‐mut 5KR, with or without the treatment of CBP inhibitor, were lysed and immunoprecipitated using anti‐Myc antibody, followed by detection of Pan‐KLA. All immunoblots had three biological repetitions, with similar results.

We first confirmed that PARP1 is a lactylated protein by co‐immunoprecipitation (Co‐IP) assays (Figure 1A,B). To identify the potential enzymatic mechanism of PARP1 lactylation, we tested enzymes, including CBP, which was previously shown to catalyze the lactylation of MRE11 as a lactyltransferase,^25^ as well as other lactyltransferases, including p300, GCN5, and PCAF. We found that CBP primarily mediated PARP1 lactylation (Figure S1A,B). Moreover, the in vitro lactylation assay further confirmed that CBP is the lactyltransferase for PARP1 lactylation (Figure 1C). We further suggested the interaction of CBP with PARP1 (Figure 1D,E). Moreover, CBP activation results in obviously increased PARP1 lactylation, while CBP inhibition leads to sharply decreased PARP1 lactylation (Figure 1F,G; Figure S1C,D).

To identify the potential PARP1 lactylation sites, Co‐IP assays suggested that the sites are mainly in amino acids 203‐476 of PARP1. As the lactylation level in amino acids 1‐203 of PARP1 is sharply decreased compared with that in amino acids 1‐476 of PARP1 (Figure 1H; Figure S1E). Moreover, CBP overexpression significantly enhanced the lactylation level in amino acids 203‐476 of PARP1, but not in amino acids 1‐203 or 484‐1014 of PARP1 (Figure S1F). Besides, doxorubicin (DOX) treatment induced the most pronounced increase in lactylation within the amino acids 203‐476 of PARP1. In contrast, DOX treatment did not appreciably elevate lactylation levels of the amino acids 1‐203 or 484‐1014 of PARP1 (Figure S1G). In addition, lactylation site mass spectrometry analysis, which screens each potential lysine to identify lactylation sites for a specific protein, was used to identify the lysine residues on PARP1 that are lactylated. The results further indicated that K203, K209, K221, K337, and K425 are all potential lactylation sites of the PARP1 protein (Figure 1I). Therefore, lysine (K) K203, K209, K221, K337, and K425 were replaced by arginine (R) to construct a 5KR mutant of PARP1. The degree of PARP1 lactylation increased with CBP overexpression and decreased with CBP inhibition, while the PARP1 mutant 5KR lactylation remained low, regardless of CBP expression levels (Figure 1J,K; Figure S1H,I). Altogether, these results suggest that CBP is the lactyltransferase for PARP1 lactylation, and the K203, K209, K221, K337, and K425 sites may be physiologically relevant lactylation sites.

### 2.2 SIRT1 is the delactylase for PARP1

**FIGURE 2:**
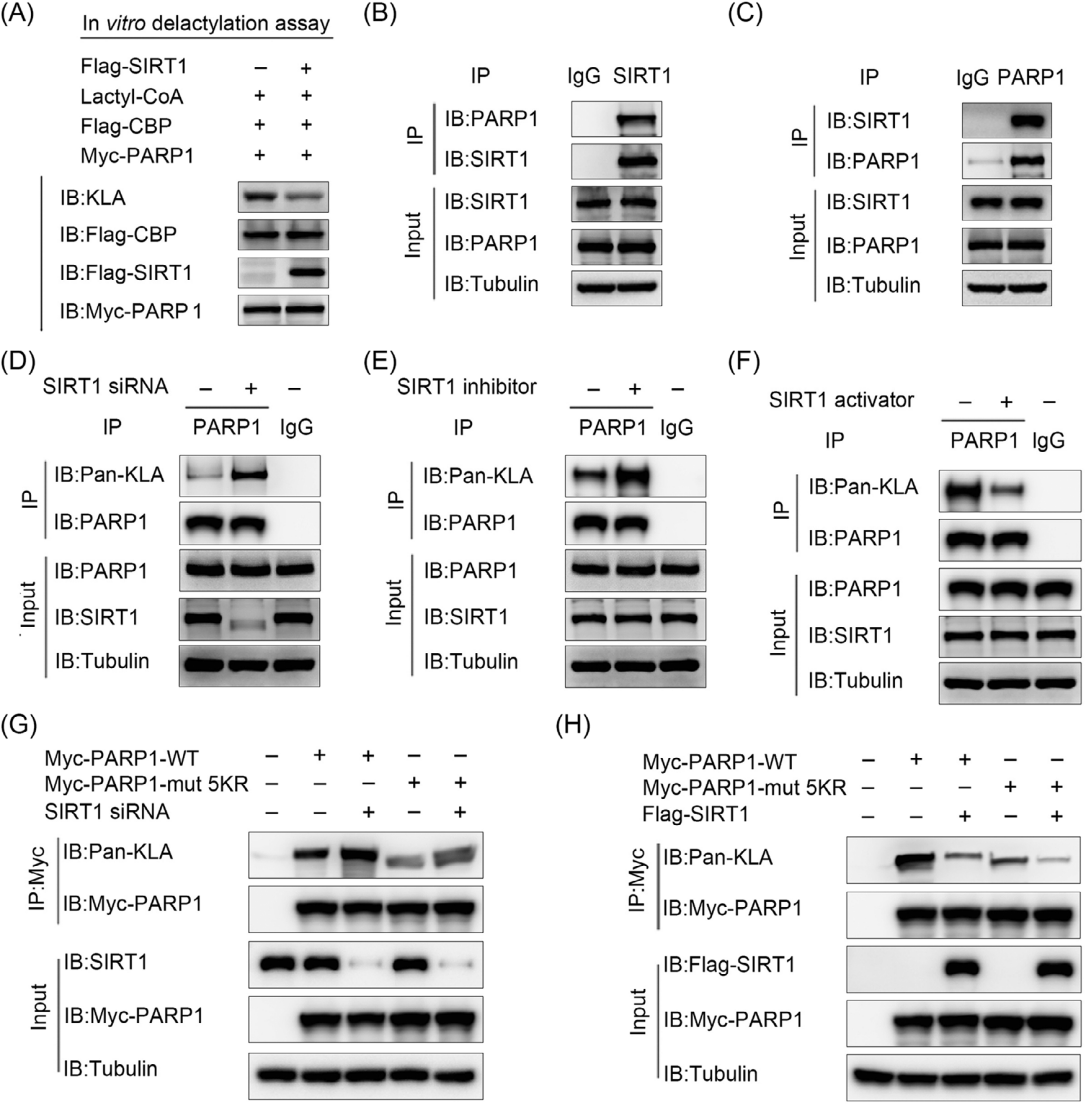
SIRT1 is the delactylase for PARP1. (A) SIRT1 can specifically erase lactylation of PARP1 in vitro. (B, C) Co‐IP of endogenous proteins from HL‐1 cell lysates using anti‐SIRT1 or Anti‐PARP1 antibody, followed by immunoblot of SIRT1, PARP1 and Tubulin. (D–F) HL‐1 cells were transfected with SIRT1 siRNAs or treated with SIRT1 inhibitor or SIRT1 activator. IP of endogenous proteins from HL‐1 cell lysates was performed using anti‐PARP1 antibodies, followed by detection of Pan‐KLA. (G) HL‐1 cells transfected with Myc‐PARP1‐WT, Myc‐PARP1‐mut 5KR and SIRT1 siRNA were lysed and immunoprecipitated using anti‐Myc antibody, followed by detection of Pan‐KLA (*n* =  3 per group). (H) HL‐1 cells transfected with Myc‐PARP1‐WT and Myc‐PARP1‐mut 5KR and Flag‐SIRT1 were lysed and immunoprecipitated using anti‐Myc antibody, followed by detection of Pan‐KLA. All immunoblots had three biological repetitions, with similar results.

To further clarify the regulatory mechanism, we aim to identify a relevant PARP1 delactylase. Given that Sirtuin family proteins are crucial deacetylases,^29^ we selected the Sirtuin family enzymes (SIRT1‐7) as candidate proteins for delactylazing PARP1. We found that only the expression of SIRT1 significantly reduced the degree of PARP1 lactylation (Figure S2A,B). Moreover, in vitro delactylation assay further confirmed that SIRT1 is the delactylase for PARP1 (Figure 2A). Next, we confirmed the interaction of SIRT1 and PARP1 by Co‐IP assays (Figure 2B,C). Moreover, transient silencing of SIRT1 by siRNAs or SIRT1 inhibitor resulted in increased PARP1 lactylation, while SIRT1 activator induced decreased PARP1 lactylation (Figure 2D–F; Figure S2C–E). The degree of PARP1 lactylation increased with transient silencing of SIRT1 and decreased with SIRT1 overexpression, while the PARP1 mutant 5KR lactylation remained low, regardless of SIRT1 expression levels (Figure 2G,H; Figure S2F,G). Thus, these results demonstrate that SIRT1 is capable of delactylating PARP1.

### 2.3 LDHA regulates lactate production and PARP1 lactylation

**FIGURE 3:**
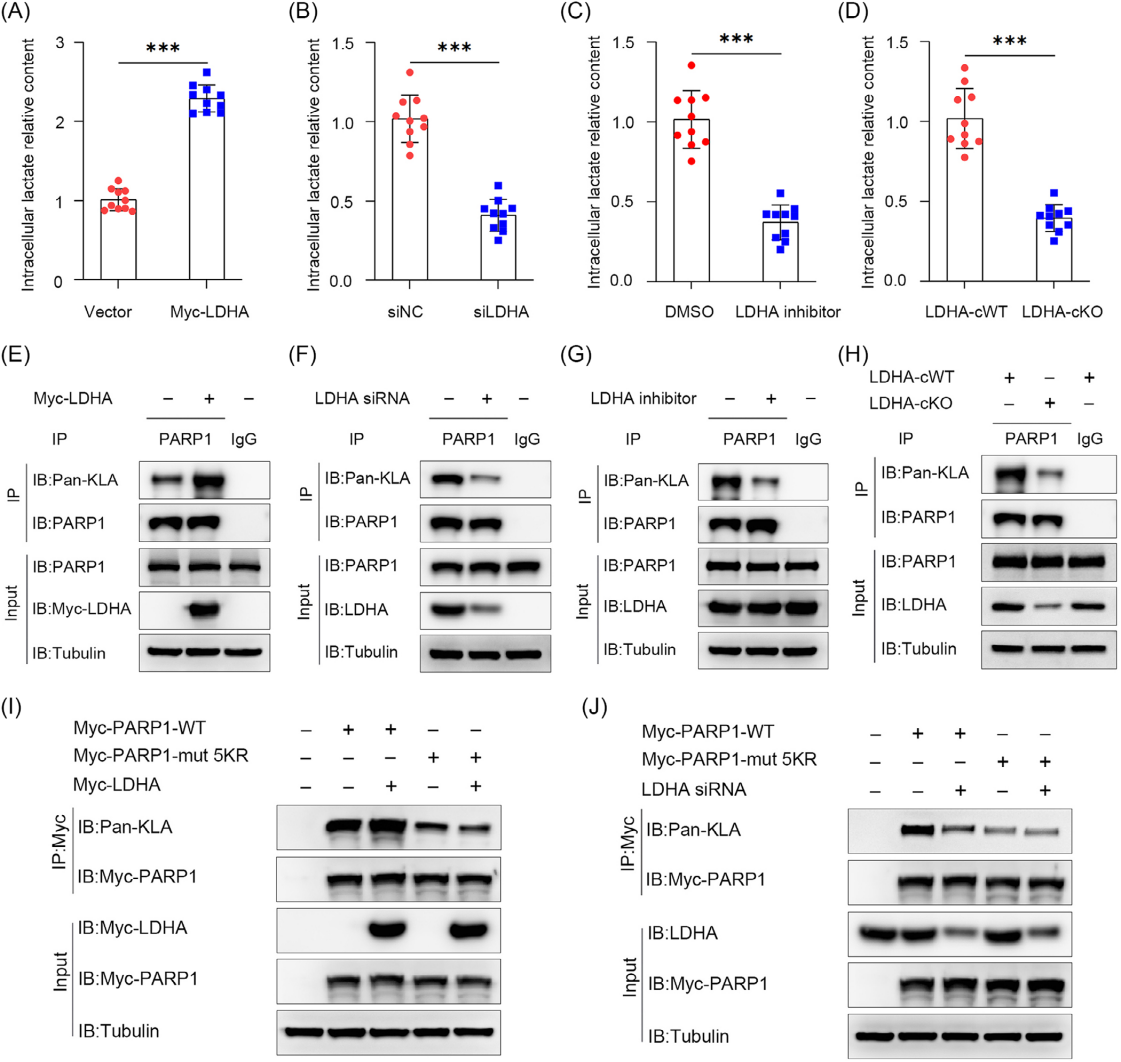
LDHA regulates lactate production and PARP1 lactylation. (A–C) Relative intracellular lactate content of HL‐1 cells after transfection with Myc‐LDHA or LDHA siRNA or LDHA inhibitor treatment (*n* = 10 per group). (D) Relative intracellular lactate content in myocardial tissues of LDHA‐cKO and LDHA‐cWT mice (*n* = 10 per group). (E–G) HL‐1 cells were transfected with Myc‐LDHA or LDHA siRNA or LDHA inhibitor treatment, and endogenous proteins in HL‐1 cell lysates were immunoprecipitated using anti‐PARP1 antibody, followed by detection of Pan‐KLA. (H) Cardiomyocytes from LDHA‐cWT or LDHA‐cKO mice were lysed and immunoprecipitated with anti‐PARP1 antibody, followed by detection of Pan‐KLA. (I) HL‐1 cells transfected with Myc‐PARP1‐WT and Myc‐PARP1‐mut 5KR and Myc‐LDHA were lysed and immunoprecipitated using anti‐Myc antibody, followed by detection of Pan‐KLA. (J) HL‐1 cells transfected with Myc‐PARP1‐WT and Myc‐PARP1‐mut 5KR and LDHA siRNA were lysed and immunoprecipitated using anti‐Myc antibody, followed by detection of Pan‐KLA. Based on the central limit theorem, the data were considered to be normally distributed. Relative protein levels were calculated as fold changes vs. the first group. Data are presented as mean ± SD. (A–D) The unpaired *t*‐test (two‐tailed Student *t*‐test) was used to compare 2 groups. All immunoblots had three biological repetitions, with similar results. ***p < .001.

LDHA is the major enzyme mediating lactate production in cells,^30^ which may, in turn, promote protein lactylation.^24^ Therefore, we speculate that LDHA may act as another important regulatory factor to maintain the degree of PARP1 lactylation. Indeed, lactate concentration increased with LDHA overexpression and decreased with the transient silencing of LDHA or application of LDHA inhibitor (Figure 3A–C). Furthermore, using myocardium‐specific LDHA knockout mice (LDHA‐cKO mice), we also observed a decreased lactate concentration in myocardial tissue (Figure 3D). As expected, the degree of PARP1 lactylation increased with LDHA overexpression and decreased with the transient silencing of LDHA, application of LDHA inhibitor, as well as in LDHA‐cKO mice (Figure 3E–H; Figure S3A–D). Moreover, the degree of PARP1 lactylation increased with LDHA overexpression and decreased with transient silencing of LDHA, while the PARP1 mutant 5KR lactylation remained low, regardless of LDHA expression levels (Figure 1I,J; Figure S3E,F). Collectively, these findings suggest that LDHA regulates PARP1 lactylation by altering lactate production.

### 2.4 ROS‐ATM‐CBP signalling pathway activates PARP1 lactylation

**FIGURE 4:**
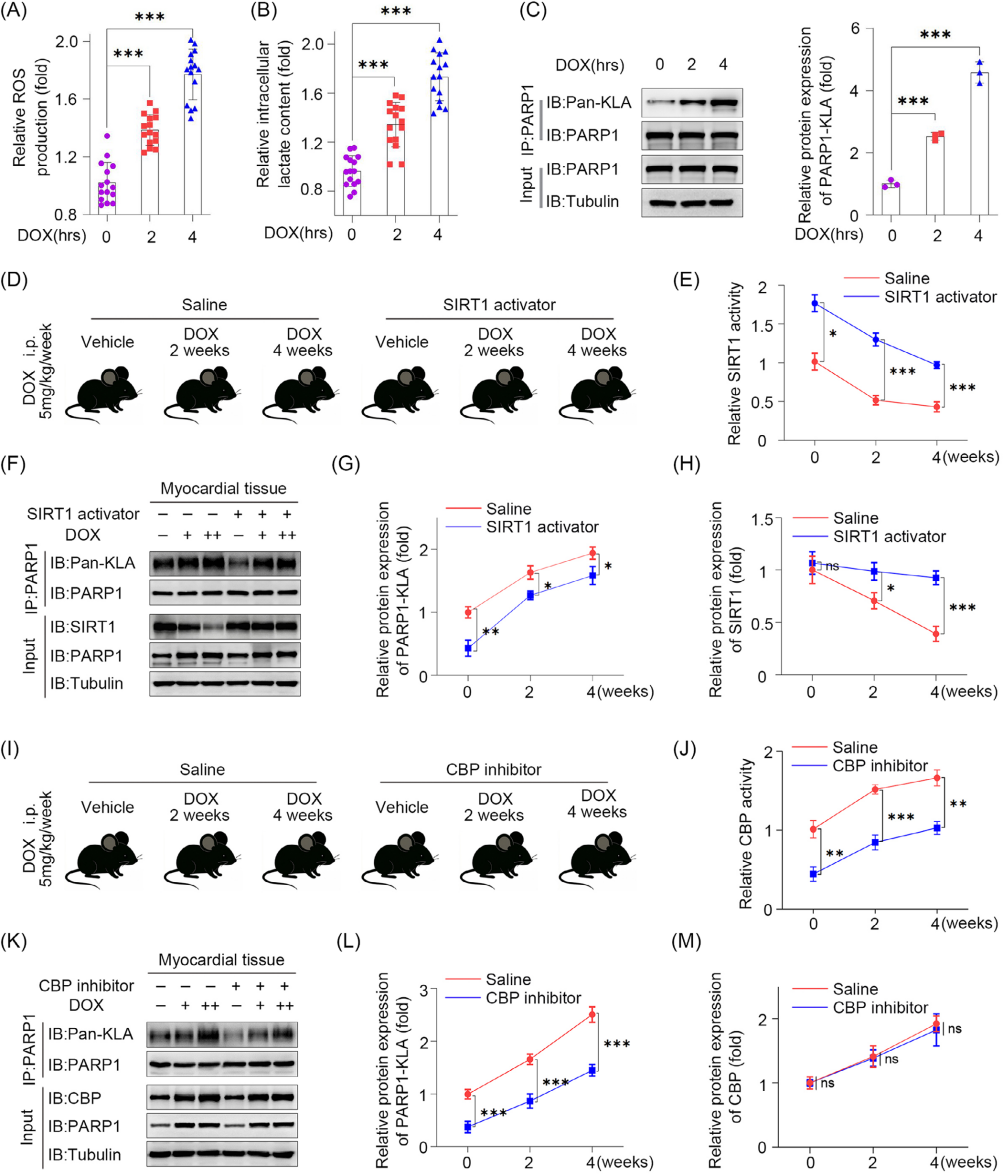
ROS‐ATM‐CBP signalling pathway activates PARP1 lactylation. (A, B) Intracellular ROS and lactate levels in HL‐1 cells treated with 1 µM doxorubicin for 0, 2 and 4 h. (C) Representative immunoprecipitation, western blots and quantification for detecting PARP1 lactylation. HL‐1 cells were stimulated by doxorubicin (1 µM for 0, 2 and 4 h), and the cells were lysed and immunoprecipitated using an anti‐PARP1 antibody, followed by detection of Pan‐KLA. (D) Schematic of the effect of SIRT1 activation on the lactylation level of PARP1 in mice. DOX (5 mg/kg) or the same volume of saline was administered intraperitoneally once weekly. One week before the DOX treatment, mice were pre‐treated with resveratrol (SIRT1 activator, 10 mg/kg/day) via daily intraperitoneal injections. (E) The SIRT1 activity was detected. (F–H) Representative immunoprecipitation, western blots and quantification for detecting PARP1 lactylation and protein expression of SIRT1. (I) Schematic of the effect of CBP inhibition on the lactylation level of PARP1 in mice. DOX (5 mg/kg) or the same volume of saline was administered intraperitoneally once weekly. One week before the DOX treatment, mice were pre‐treated with SGC‐CBP30 (CBP inhibitor, 15 mg/kg/day) via daily intraperitoneal injections. (J) The CBP activity was detected. (K–M) Representative immunoprecipitation, western blots and quantification for detecting PARP1 lactylation and protein expression of CBP. (A–C) Based on the central limit theorem, the data were considered to be normally distributed. Relative protein levels were calculated as fold changes vs the first group. Data are presented as mean ± SD. Statistical significance was assessed by one‐way ANOVA with Tukey multiple comparisons test (*P* values adjusted for 3 comparisons). (D–M) Relative protein levels were calculated as fold changes vs. the first group. Data are presented as mean ± SD. Statistical significance was assessed by two‐way ANOVA with Bonferroni multiple comparisons test (*p*‐values adjusted for 9 comparisons). ns *p *> .05, *p < .05, **p < .01, ***p < .001.

Excessive oxidative stress induces the accumulation of ROS and altered metabolic features.^31^ We observed that DOX led to an increase in ROS production and lactate accumulation (Figure 4A,B). Since lactate enables the lysine lactylation of proteins,^24^ we further examine the changes of PARP1 lactylation in response to oxidative stress. First, we find that DOX induced a gradual increase in PARP1 lactylation with prolonged stimulation (Figure 4C). Similarly, ROS induced by H_2_O_2_ also present a gradual increase in the PARP1 lactylation with prolonged stimulation (Figure S4A). Moreover, when we treated cells with the antioxidant Tempol and found that the PARP1 lactylation induced by DOX was reduced (Figure S4B). This result indicates that ROS are key signal molecules to induce PARP1 lactylation.

Our previous studies have shown that the acetyltransferase CBP and deacetylase SIRT2 regulated PARP1 acetylation, which dictates oxidative stress‐induced vascular injury.^19^ In our study, CBP and SIRT1 work as lactyltransferase and delactylase of PARP1, respectively. We therefore wondered if PARP1 lactylation depends on the lactyltransferase CBP or delactylase SIRT1 under oxidative stress. However, despite effectively preserving SIRT1 expression and activity under DOX treatment, we found that the SIRT1 activator merely partially attenuated the DOX‐induced increase in PARP1 lactylation, but the effect of the SIRT1 activator was not as pronounced as the CBP inhibitor in DOX‐induced cardiotoxicity (Figure 4D–M). As noted, DOX leads to decreased expression and activity of SIRT1 in the myocardium (Figure 4E,H), which may contribute to increased PARP1 lactylation. Nevertheless, the interaction between SIRT1 and PARP1 was not reduced but remained unchanged (Figure S4C,D), suggesting that SIRT1 may have attempted yet failed to fully prevent the increase in PARP1 lactylation. In contrast, CBP expression and activity were significantly increased (Figure 4J,M), and its interaction with PARP1 was markedly enhanced under DOX treatment (Figure S4E,F). Moreover, consistent results were also obtained in rat primary cardiomyocytes, where SIRT1 overexpression only partially attenuated the DOX‑induced increase in PARP1 lactylation, and its effect was markedly weaker than that of CBP knockdown under DOX stimulation (Figure S4G,H). Therefore, we proposed that CBP may be a more crucial player than SIRT1 in the regulation of PARP1 lactylation under DOX stimulation.

As a sensor for the intracellular ROS signalling pathway, ATM is typically phosphorylated at S1981 under oxidative stress.^32^ Current study has also demonstrated that the phosphorylated ATM further phosphorylates CBP to positively regulate the lactylation of MRE11.^25^ Therefore, to further confirm the requirement for ATM in PARP1 lactylation, we investigated the effect of inhibition of ATM activity on PARP1 lactylation. The application of a pharmacological ATM inhibitor diminished the PARP1 lactylation increase as well as the interaction of CBP with the PARP1 increase upon oxidative stress (Figure S4I,J). These results indicated that the ATM‐CBP pathway serves as a sensitive and specific sensing mechanism that uses intracellular ROS to stimulate PARP1 lactylation.

### 2.5 CBP exacerbates DOX‑induced myocardial injury primarily through the PARP1 lactylation–PARylation axis

PARP1 is an abundant nuclear protein involved in cell death pathways. Previous studies have shown that PARP1 promotes cardiac dysfunction via PARylation of downstream targets, including BRD4, HMGB1, CEBP/β, and FOXO3a.^33^, ^34^, ^35^, ^36^ In our study, we identified CBP as a crucial player in the regulation of PARP1 lactylation under DOX stimulation. Given that CBP serves as a broad transcriptional and epigenetic regulator with multiple downstream targets, we therefore wondered if CBP could exacerbate DOX‑induced myocardial injury mainly through the PARP1 lactylation–PARylation axis in rat primary cardiomyocytes (Figure S5A).

**FIGURE 5:**
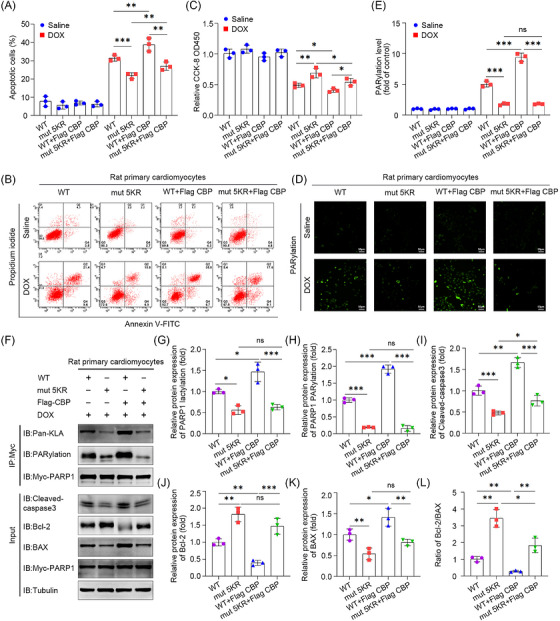
CBP exacerbates DOX‑induced myocardial injury primarily through the PARP1 lactylation–PARylation axis. (A, B) Representative flow cytometry analysis and quantitative analysis of apoptosis in rat primary cardiomyocytes induced by DOX (1 µM for 24 h). (C) The CCK‐8 assay was used to assess the viability of rat primary cardiomyocytes. (D, E) Representative PARylation staining and quantitative analysis induced by DOX (1 µM for 24 h) in rat primary cardiomyocytes. (F–L) Representative western blots and quantification of PARP1 lactylation/PARylation, as well as apoptosis marker (cleaved‐caspase 3, Bcl‐2 and BAX) in rat primary cardiomyocytes under DOX (1 µM for 24 h) treatment. Based on the central limit theorem, the data were considered to be normally distributed. Relative protein, apoptosis, viability and PARylation levels of rat primary cardiomyocytes were calculated as fold changes vs. the first group. Data are presented as mean ± SD. (A, C, E, G–L) Statistical significance was assessed by one‐way ANOVA with Tukey multiple comparisons test (*p*‐values adjusted for 6 comparisons). All immunoblots had three biological repetitions, with similar results. ns *p *> .05, *p < .05, **p < .01, ***p < .001.

Under DOX stimulation, the PARP1 mutant 5KR significantly reduced cardiomyocyte apoptosis compared with the PARP1 WT group. Overexpression of Flag‑CBP markedly aggravated apoptosis in the PARP1 WT group. However, this pro‐apoptotic effect was substantially blunted in the PARP1 mutant 5KR group, where Flag‐CBP overexpression induced only a marginal increase in apoptosis (Figure 5A,B). Using an independent sensitive colourimetric assay, we found that the PARP1 mutant 5KR restored DOX‑impaired cell proliferation and viability compared with the PARP1 WT group. Flag‑CBP overexpression strongly reduced cell viability in the PARP1 WT group under DOX stimulation. In contrast, this suppressive effect mediated by CBP was markedly attenuated in the PARP1 mutant 5KR group, where only a partial reduction in viability was observed (Figure 5C). Moreover, we first investigated the effect of PARP1 lactylation on PARP1 PARylation. Surprisingly, immunofluorescence staining and western blotting consistently showed that, under DOX stimulation, the PARP1 mutant 5KR dramatically reduced the expression level of cellular total PARylation, PARP1 lactylation/PARylation, and cleaved caspase‐3, as well as increased the ratio of Bcl‐2/BAX. Overexpression of Flag‑CBP robustly enhanced these post‐translational modifications and pro‐apoptotic phenotypes in the PARP1 WT group. Notably, these CBP‐mediated effects were largely abrogated in the PARP1 mutant 5KR group, where only partial and negligible alterations were observed (Figure 5D–L).

To further explore the specific mechanisms of the lactylation–PARylation cascade in apoptosis. The PARylation of downstream targets and the interaction between PARP1 WT/PARP1 mutant 5KR with them were tested. As expected, the PARylation of BRD4, HMGB1, CEBP/β, and FOXO3a increased under the DOX treatment in the PARP1 WT group, while the PARylation of BRD4, HMGB1, CEBP/β, and FOXO3a remained low, regardless of DOX treatment in the PARP1 mutant 5KR group (Figure S5B,G). Besides, the interaction between PARP1 WT with BRD4, HMGB1, CEBP/β, and FOXO3a increased under the DOX treatment, while the interaction between PARP1 mutant 5KR with BRD4, HMGB1, CEBP/β, and FOXO3a remained weak, regardless of DOX treatment (Figure S5C–F,H).

Taken together, while we acknowledge that CBP may regulate lactylation or other modifications of additional molecules, our data suggest that CBP might exacerbate DOX‐induced myocardial injury mainly through promoting the PARP1 lactylation–PARylation axis. The PARP1 mutant 5KR, which specifically abolishes PARP1 lactylation, largely rescues the detrimental effects of CBP overexpression. These findings might establish a causal relationship between PARP1 lactylation and DOX‐induced myocardial injury, with CBP acting as a critical upstream regulator.

### 2.6 Myocardium‐specific CBP knockout mice decrease PARP1 lactylation and prevent doxorubicin‐induced cardiac dysfunction

**FIGURE 6:**
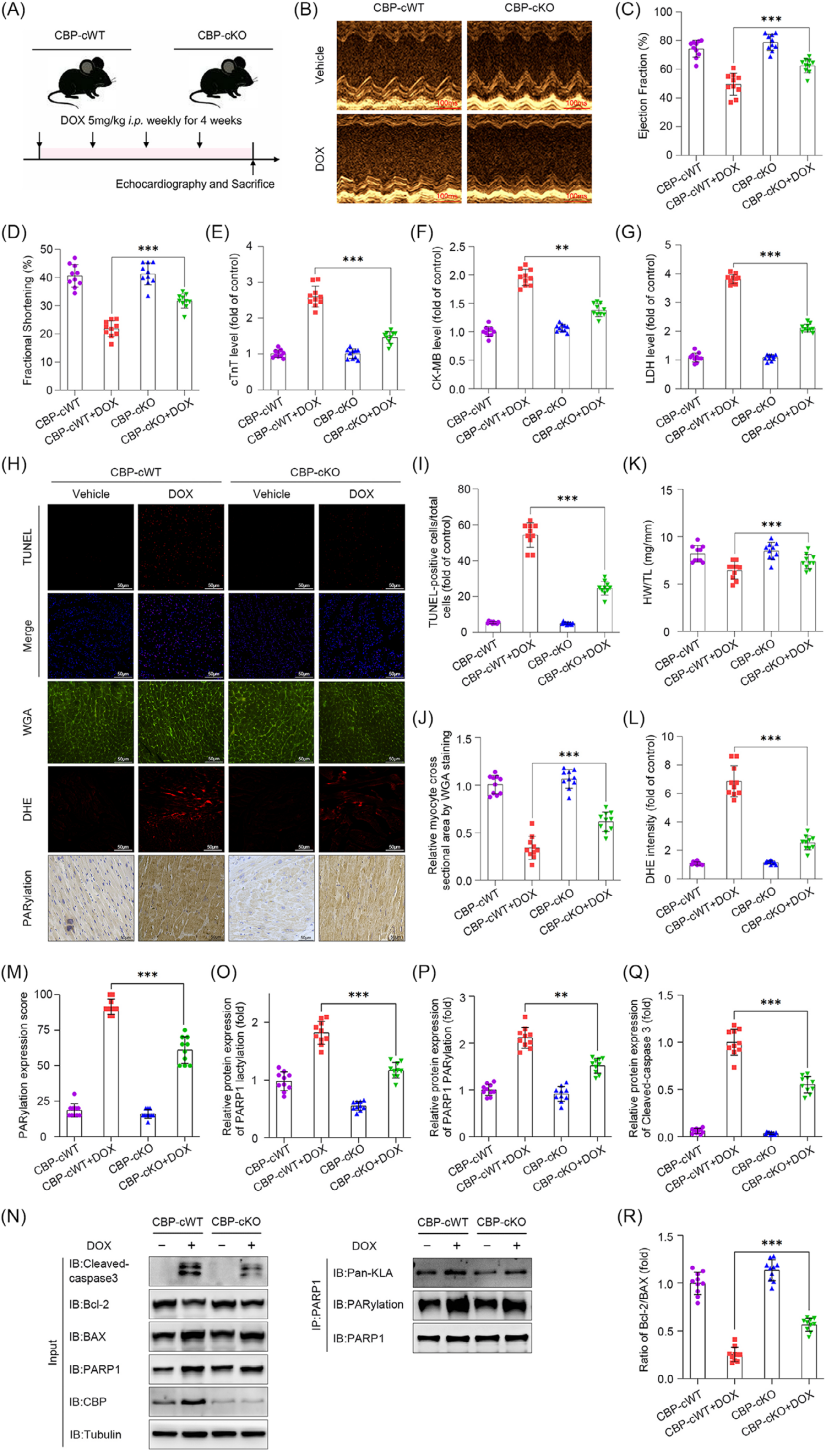
Myocardium‐specific CBP knockout mice decrease PARP1 lactylation and prevent doxorubicin‐induced cardiac dysfunction. (A) Schematic of DOX‐induced cardiac dysfunction in CBP‐cKO mice and CBP‐cWT mice. DOX was intraperitoneally injected once a week with a dose of 5 mg/kg or the same volume of saline for 4 weeks (20 mg/kg cumulative dose of doxorubicin). (B–D) Representative echocardiography and quantitative analysis of EF% and FS% (*n* = 10 per group). (E–G) Quantitative analysis of Serum cTnT, CK‐MB and LDH levels (*n* = 10 per group). (H) Representative TUNEL staining, WGA staining, DHE staining and immunohistochemistry staining (*n* = 10 per group). (I) Quantification of apoptotic cardiomyocytes by TUNEL staining (*n* = 10 per group). (J) Quantification of relative cardiomyocyte cross‐sectional areas by WGA staining (*n* = 10 per group). (K) The ratio of heart weight to tibia length (HW/TL, mg/mm) (*n* = 10 per group). (L) Quantification of DHE staining intensity (*n* = 10 per group). (M) Quantification of total PARylation by immunohistochemistry staining (*n* = 10 per group). (N–R) Representative immunoprecipitation, western blots and quantification of PARP1 lactylation/ PARylation as well as apoptosis marker (cleaved‐caspase 3, Bcl‐2 and BAX) (*n* = 10 per group). (C–G, I–M, O–R) Relative protein levels were calculated as fold changes vs. the first group. Data are presented as mean ± SD. Statistical significance was assessed by two‐way ANOVA with Bonferroni multiple comparisons test (*p*‐values adjusted for 6 comparisons). ns *p *> .05, *p < .05, **p < .01, ***p < .001.

In our study, we found that CBP could exacerbate DOX‐induced myocardial injury mainly through promoting the PARP1 lactylation–PARylation axis, and abolishing PARP1 lactylation largely rescues the detrimental effects of CBP overexpression. Therefore, we further employed myocardium‐specific CBP knockout (CBP‐cKO) mice to investigate the role of CBP‐PARP1 lactylation–PARylation axis in doxorubicin cardiotoxicity (Figure 6A).

Notably, CBP‐cKO mice displayed significantly alleviated doxorubicin‐induced cardiac dysfunction, as reflected by preserved ejection fraction (EF%) and fractional shortening (FS%) (Figure 6B–D). Myocardial injury was also assessed by the serum levels of cTnT, CK‐MB and LDH, which indicated that CBP‐cKO mice could alleviate doxorubicin‐induced myocardial injury (Figure 6E–G). Myocardial apoptosis was also alleviated in CBP‐cKO mice as revealed by TUNEL staining (Figure 6H,I). Furthermore, CBP‐cKO mice preserved the size of cardiomyocytes under the treatment of doxorubicin (Figure 6H,J). The ratio of heart weight to the tibial length (HW/TL) was decreased in the doxorubicin group, which was significantly blocked in CBP‐cKO mice (Figure 6K). Besides, the CBP‐cKO mice decreased the ROS level under the treatment of doxorubicin as revealed by DHE staining (Figure 6H,L). Moreover, doxorubicin‐treated mice had increased levels of PARP1 lactylation/PARylation and cellular total PARylation in myocardium tissues compared with the control group, while CBP‐cKO significantly reduced certain changes (Figure 6H,M–P). Furthermore, under DOX stimulation, CBP‑cKO mice decreased cleaved caspase‑3 expression and increased Bcl‑2/BAX ratio compared with CBP‑cWT mice (Figure 6N,Q,R).

Last but not least, we further evaluate whether CBP inhibition (SGC‑CBP30) compromises the antitumour efficacy of doxorubicin. As shown in Figure S6A–D, co‑treatment with CBP inhibitor further reduced cell viability compared with DOX treatment alone across a panel of cancer cell lines, including MV4‑11 (acute leukaemia), SKOV3 (ovarian cancer), MDA‑231 (triple‑negative breast cancer), and Raji (Burkitt's lymphoma). Moreover, in MV4‑11 cells, CBP inhibitor co‑treatment significantly decreased the mRNA levels of the oncogene MYC, cell‐cycle regulators CCNB1 and CDK1, and the anti‑apoptotic protein BIRC5 compared with DOX treatment alone (Figure S6E–H). Taken together, these results suggest that CBP inhibition might not impair, but rather enhance the antitumour efficacy of doxorubicin by suppressing oncogenic signalling and sensitizing cancer cells to chemotherapy.

Collectively, these in vivo and in vitro experiments support that CBP could exacerbate DOX‑induced cardiotoxicity mainly through enhancing the PARP1 lactylation–PARylation axis, and that the inhibition of CBP confers substantial cardiac protection and preserves or even augments the antitumour activity of DOX.

## 3 DISCUSSION

**FIGURE 7:**
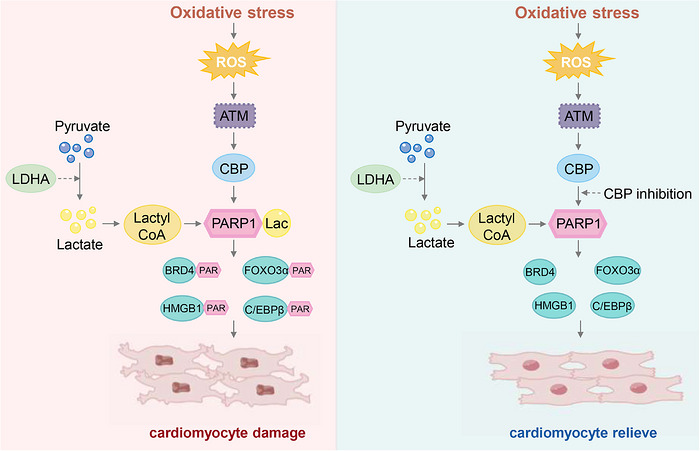
Model showing the molecular signalling cascade of CBP‐PARP1 lactylation–PARylation axis. The schematic depicts how oxidative stress‐induced ROS activates the ATM‐CBP‐PARP1 signalling pathway via a lactylation–PARylation cascade to promote cardiomyocyte apoptosis.

In this study, we found that the ROS‐ATM‐CBP axis is activated in response to oxidative stress, which mediates the PARP1 lactylation–PARylation cascade and induces apoptosis. Moreover, inhibiting the overactivation of PARP1 by Myocardium‐specific CBP knockout could alleviate doxorubicin‐induced cardiac dysfunction (Figure 7). These findings demonstrate the detailed molecular mechanisms by which PARP1 lactylation is induced for subsequent apoptosis and myocardial injury.

PARP1 acts as a critical DNA damage sensor and epigenetic transcriptional regulator, rapidly detecting genomic lesions to initiate DNA repair and dynamically remodelling chromatin structure to modulate gene expression. These biological functions rely strictly on its NAD^+^‑dependent PARylation activity, which generates branched PAR chains onto itself or target proteins. The resulting PAR scaffolds serve as a protein‐recruitment platform for PAR‐binding effectors and as a functional switch to reshape target protein conformation, activity, or interactions.^37^ In DNA damage repair, its N‐terminal zinc fingers sense damage, trigger intense auto‐PARylation, and recruit DNA repair factors such as XRCC1,^38^, ^39^, ^40^ DNA ligase III,^38^, ^41^ MRE11,^42^, ^43^ ALC1^44^, ^45^ and p53,^46^, ^47^ thereby orchestrating efficient base excision repair and stalled replication fork stabilization. PARP1‐mediated histone PARylation relaxes chromatin architecture to facilitate lesion accessibility for repair machinery,^48^ and PARG‐dependent PAR degradation ensures timely termination of DNA damage signalling upon repair completion.^49^ Beyond DNA repair, PARP1 also acts as an enzyme‑independent coactivator for NF‑κB,^50^ suppresses C/EBPβ DNA‑binding activity,^51^ and modulates the transcriptional programs governed by STAT3, Erα and Sox2.^52^ PARP1 could balance DNA methylation by inhibiting DNMT1 activity via auto‑PARylation in a CTCF‑modulated manner and potentiating TET1‑dependent demethylation.^53^, ^54^ At the histone level, PARP1 displaces histone H1, restrains KDM5B‑mediated H3K4me3 demethylation, and enriches 5hmC levels to sustain stem cell proliferative and differentiation potential.^53^, ^55^ Pathologically, oxidative stress induces excessive PARP1 activation, which suppresses hexokinase 1 to disrupt glycolysis before substantial NAD^+^ depletion,^56^ subsequently impairing mitochondrial TCA cycling and oxidative phosphorylation, and ultimately causing ATP exhaustion, mitochondrial depolarization, and necrotic cell death.^57^, ^58^ Meanwhile, hyperactivated PARP1 aggravates vascular inflammation and atherogenesis by enhancing NF‑κB‑dependent proinflammatory cytokine production,^59^ promoting HMGB1 release as a damage‐associated molecular pattern (DAMP),^60^ upregulating endothelial ICAM‑1/VCAM‑1 to facilitate monocyte adhesion, and accelerating macrophage foam cell formation.^61^, ^62^ Furthermore, PARP1 competes with SIRT1 for limited intracellular NAD^+^, linking its overactivation to metabolic dysfunction and persistent vascular inflammation.^63^ Collectively, PARP1 acts as a central signalling hub that integrates DNA damage surveillance, epigenetic remodelling, energy metabolism and inflammatory responses, thereby bridging oxidative genomic injury to cardiovascular pathological progression.

Substrate‐specific acetylation catalyzed by CBP bridges epigenetic dysregulation to the onset and progression of diverse human diseases. At the histone level, CBP targets H2B, H3 and H4 to drive chromatin remodelling and transcriptional regulation;^64^ germline loss‐of‐function mutations impair this histone acetyltransferase activity and trigger Rubinstein–Taybi syndrome,^65^ while preclinical mouse models confirm that diminished global histone acetylation coincides with impaired cognitive function.^66^ For non‐histone transcription factors, CBP acetylates p53 at K320, K373 and K382 to stabilize p53 and reinforce its tumour‐suppressive capacity.^66^, ^67^, ^68^, ^69^ In breast cancer, FXR antagonizes CBP to block p53 K382 acetylation, which represses ferroptosis and facilitates tumour proliferation.^69^ Conversely, CBP overexpression fuels prostate oncogenesis partially via perturbing canonical p53 signalling.^70^ Furthermore, CBP activates Bim expression involved in CVB3‐induced cardiomyocyte apoptosis,^71^ whereas suppressed CBP activity in obesity and type 2 diabetes diminishes FOXO1 acetylation, triggering FOXO1 ubiquitination and degradation to alleviate excessive hepatic gluconeogenesis and lipogenesis.^72^ Moreover, CBP acetylates MOB1 at K11 to stabilize MOB1 and sustain Hippo signalling, which promotes YAP/TAZ turnover; reduced MOB1 acetylation correlates with unfavourable clinical outcomes and accelerated disease progression in lung adenocarcinoma, supporting a tumour‐suppressive function of this CBP modification.^73^ CBP additionally acetylates androgen receptor (AR) to protect AR from proteasomal degradation and sustain AR transcriptional signalling, and aberrantly elevated CBP activity sustains AR‐dependent oncogenic transcription in castration‐resistant prostate cancer.^70^ Broadly speaking, pathological CBP dysfunction manifests through multiple molecular mechanisms and underpins a wide range of human disorders: germline mutations causing CBP haploinsufficiency lead to Rubinstein–Taybi syndrome^65^; somatic inactivating mutations and chromosomal translocations drive acute myeloid leukaemia and B‐cell lymphomas;^74^, ^75^ mutant disease proteins functionally inhibit CBP activity in Huntington's neurodegenerative diseases.^76^ Collectively, these tissue‐specific acetylation substrates and their associated disease phenotypes establish CBP as an indispensable epigenetic rheostat. Its acetyltransferase activity maintains physiological development, cell differentiation and tissue homeostasis, whereas disrupted CBP‐mediated acetylation cascades initiate and propagate pathological processes across developmental, neoplastic and neurodegenerative diseases.

Recent studies have demonstrated that lactate can induce a new PTM called lactylation, which alters the function of histone or non‐histone proteins by modifying lysine residues.^24^ The lactylation of PARP1 was reported by a bioorthogonal chemical reporter for the detection and identification of protein lactylation.^28^ However, the signal transduction cascade and physiological function of PARP1 lactylation are unknown. Our study firstly described CBP as the key regulator in PARP1 lactylation under oxidative stress. Similarly, as a lactyltransferase for protein lactylation, CBP was also reported to positively regulate the lactylation of MRE11, Snail1 and HMGB1.^25^, ^77^, ^78^ Similarly, some usual lactyltransferases, including p300,^24^ CBP,^25^, ^77^, ^78^ GCN5,^79^ and PCAF, may be the potential lactylation writer for other proteins. The relationship of CBP with PARP1 has been suggested by several previous investigations. It has been reported that the recruitment of macroH2A1.1 by PARP1 promoted the CBP‐mediated acetylation of H2B, which positively regulated cancer suppression.^80^ The acetylation of PARP1 by p300/CBP plays an important regulatory role in NF‐κB‐dependent gene activation by enhancing its functional interaction with p300 and the mediator complex.^81^ In addition, our previous studies have also shown that the acetyltransferase CBP regulates PARP1 acetylation, which dictates oxidative stress‐induced vascular injury.^19^ In this study, we found that ROS‐induced CBP‐mediated PARP1 lactylation might turn out to be a key event in cellular response to oxidative stress.

The main histone deacetylases families in mammalians, Zn^2+^‐dependent HDAC family (HDACs 1‐3) and NAD^+^ dependent SIRT family (SIRT1‐3), were newly identified as lactylases for histone delactylation after discovering the lactylation of histones.^81^ As a delactylase for non‐histone protein delactylation, SIRT2 regulates the delactylation of METTL16 and inhibits cuproptosis via suppression of m6A‐modification on FDX1 mRNA in gastric cancer.^82^ Besides, delactylase SIRT3‐regulated mitochondrial protein delactylation (PDHA1 and CPT2) integrates intracellular hypoxia and lactate signals to regulate OXPHOS.^27^ Similarly, SIRT1 was also reported to positively regulate the delactylation of α‐MHC^83^ and HMGB1.^78^ In our study, SIRT1 is identified as the specific delactylase for PARP1, and the biological relationships between PARP1 and SIRT1 are also described in detail. SIRT1 and PARP1 were together known as epigenome safeguards and microRNAs as SASP‐associated signals, in cellular senescence and ageing.^84^ SIRT1 is also a highly conserved nuclear deacetylase and often acts as an inhibitor of parthanatos by deacetylation of PARP1.^85^ Furthermore, studies indicated that the ATPase domain of BRG1 and the ZnF domain of SIRT1 interact with PAR polymers generated by PARP1 in response to DNA damage, and are responsible for bringing the two factors to broken DNA ends.^86^ We found that SIRT1 is responsible for the PARP1 delactylation, but SIRT1 may not be the core modulator in the PARP1 lactylation under oxidative stress.

Lactate can be produced through glycolysis or glutamine metabolism, which acts on the specific receptor GPR81 to mediate biological processes such as energy metabolism, lipodieresis, and inflammatory regulation.^87^ Lactate can also be transported into cells by MCTs to achieve lactate homeostasis.^88^ The metabolism of lactate in cells occurs through two pathways. On the one hand, lactate is oxidized by pyruvate dehydrogenase (PDH) to pyruvate and metabolized through the TCA. On the other hand, it is converted to lactyl‐CoA and participates in the lactylation of histones and non‐histones.^89^ Here, we found that the inhibition of LDHA activity reduces the lactate production and inhibits PARP1 lactylation. On the contrary, overexpression of LDHA increases cellular lactate production and thereby enhances PARP1 lactylation. In addition, the relationships between lactate and cellular injury have also been described. Excessive ROS can be produced in mitochondria when lactate is actively oxidized, and may lead to severe and irreversible neuron injury. Furthermore, excessive ROS also impairs ATP synthesis, resulting in higher ROS production; this vicious cycle ultimately leads to axon degeneration.^90^ The pharmacological application of the LDHA inhibitor is neuroprotective through suppressing the inflammatory response after cerebral ischemia/reperfusion injury.^91^

The molecular mechanism by which PARP1 lactylation is induced was previously unknown. In this study, we demonstrated that ROS promotes CBP to mediate PARP1 lactylation in an ATM dependent manner, which further overactivates PARP1 by elevating its PARylation to promote cell death. PARP1 participates in various cellular processes by transferring ADP‐ribose to target proteins.^3^, ^4^, ^5^ However, due to its capacity to deplete cellular energetic pools, excessive intracellular damage generates large branched‐chain PAR polymers, which finally result in cell dysfunction and even cell death.^3^, ^6^, ^7^, ^8^ This is consistent with the established concept that, depending on the severity of DNA damage, mild PARP1 activation is protective, whereas uncontrolled NAD^+^ cleavage by PARP1 is proapoptotic.^92^ Therefore, PARP1 activation intensity may be a key factor in regulating cell death or survival after oxidative stress. Current studies have also indicated that inhibiting PARP1 overactivation by suppressing cellular PARylation alleviates myocardial injury, as proved by multiple pharmacological methods.^9^, ^10^, ^11^, ^12^, ^13^ Here, we suggested that CBP inhibition demonstrates decreased PARP1 lactylation and PARylation as well as an alleviated injury phenotype of doxorubicin‐induced cardiac dysfunction.

In summary, we found that excessive ROS triggers PARP1 lactylation via the ATM‐CBP axis, which dictates PARylation of PARP1 and its substrates to induce apoptosis under oxidative stress. These findings unravelled how PARP1 lactylation leads to cell death upon oxidative stress, inhibition of which might serve as a novel approach to alleviate doxorubicin‐induced cardiac dysfunction.

## 4 MATERIAL AND METHODS

### 4.1 Cell culture

Rat primary cardiomyocytes were purchased from Cellverse and cultured in rat primary cardiomyocyte culture medium (iCell‐c001‐002r, Cellverse). HL‐1 cells were purchased from the Cell Bank in the Chinese Academy of Sciences and cultured in high‐glucose Dulbecco's modified Eagle's medium (DMEM). Human acute leukaemia (MV4‐11), ovarian cancer (SKOV3), breast cancer (MDA‐231) and Burkitt's lymphoma (Raji) were grown in RMPI or DMEM/F12 medium (Thermo Fisher Scientific) supplemented with 10% FBS (Thermo Fisher Scientific). All media described above were supplemented with 10% fetal bovine serum (FBS) (CLARK), penicillin (100U), and streptomycin (100 g/mL) before culturing cells.

### 4.2 Animals

Eight‐week‐old C57BL/6 mice were housed in pathogen‐free and temperature‐controlled conditions (22–25 ± 1°C) with a 12 h light/dark cycle and free access to food and water. The animals were randomly assigned to experimental groups. To clarify the main in vivo mechanisms by which CBP and SIRT1 dynamically regulate PARP1 lactylation in doxorubicin‐induced myocardial injury, doxorubicin (5 mg/kg) or the same volume of saline was administered intraperitoneally once weekly. One week before the doxorubicin treatment, mice were pre‐treated with either resveratrol (SIRT1 activator, 10 mg/kg/day) or SGC‐CBP30 (CBP inhibitor, 15 mg/kg/day) via daily intraperitoneal injections. Myocardium‐specific CBP knockout (CBP‐cKO) mice were employed to investigate the role of CBP‐PARP1 lactylation–PARylation axis in doxorubicin‐induced cardiac injury. Doxorubicin was intraperitoneally injected once a week with a dose of 5 mg/kg or the same volume of saline for 4 weeks (20 mg/kg cumulative dose of doxorubicin). Cardiac function was assessed by echocardiography. Mice were then sacrificed for assessment of doxorubicin‐induced cardiac injury. CBP‐cKO mice and LDHA‐cKO mice were designed and generated by Shanghai Model Organisms Center, Inc. CBP and LDHA knockout was induced by intraperitoneal injection of 50 mg/kg tamoxifen for 3 consecutive days. All animal experiments were approved and performed in accordance with the Guide for the Care and Use of Laboratory Animals of the National Institutes of Health (NIH) and the guidelines of the institutional Animal Care and Use Committee (IACUC) of China Medical University.

### 4.3 Immunofluorescence assays

Immunofluorescence detection was performed on frozen myocardial tissue sections and rat primary cardiomyocytes. The cross‐sectional area of cardiomyocytes was evaluated from images obtained after staining with 5 µM wheat germ agglutinin (WGA) (Thermo, USA). The total PARylation level of rat primary cardiomyocytes was evaluated by staining with anti‐Poly/Mono‐ADP Ribose (Cell Signaling Technology). Additionally, TUNEL staining was performed using the TUNEL apoptosis detection kit (KTA2011, Abbkine) following the manufacturer's instructions. The percentage of apoptotic cardiomyocytes was calculated by determining the ratio of the number of TUNEL‐positive cells to the total number of DAPI‐positive cells in the myocardial tissue.

### 4.4 Analysis of oxidative stress markers

In the immunofluorescence way, intracellular oxidative stress levels in myocardial tissues were detected using dihydroethidium (DHE) (C1300‐2, Applygen Technologies). After independent treatment, frozen myocardial tissue sections were washed three times with phosphate‐buffered saline (PBS), incubated with 10 µM DHE for 1 h at 37°C, and protected from light. Sections were washed 3 times with PBS and then imaged by a fluorescence microscope (Olympus) under 595 nm excitation.

In flow cytometry way, the collected HL‐1 cells were washed with PBS three times before incubating with 20 µM 2,7‐DCFH‐DA for 30 min at 37°C. Then the samples were collected in a centrifuge tube, washed with PBS and centrifuged at 1500 rpm for 5 min. We measured relative fluorescence intensity at the FITC channel by flow cytometry.

### 4.5 Echocardiography

Cardiac function was assessed with a Visual Sonics Vevo 2100 real‐time high‐resolution intravital microscopy system (Visualsonic Inc.). Mice were anesthetized with 1.5% isoflurane, and cardiac function was measured using a 40 MHz transducer under continuous oxygenation. Two‐dimensional M‐mode recordings were used to assess left ventricular function (LVEF). Cardiac function was determined by measuring interventricular septal dimensions, left ventricular posterior wall dimensions, systolic internal left ventricular dimensions, diastolic internal left ventricular dimensions, and left ventricular mass. In addition, left ventricular ejection fraction (EF%) and fractional shortening (FS%) were calculated.

### 4.6 Antibodies and reagents

The following antibodies were used for Western blotting: anti‐PARP1 (#9532, CST), anti‐CBP (#7389, CST), anti‐SIRT1 (#No.13161‐1‐AP, Proteintech), anti‐α‐tubulin (#No.11224‐1‐AP, Proteintech), anti‐Flag (#GNI4110‐FG‐M, GNI), anti‐Myc (#GNI4110‐MC‐M, GNI), anti‐LDHA (#No.19987‐1‐AP, Proteintech), anti‐Pan Kla (#PTM‐1401RM, PTMBIO), anti‐cleaved‐Caspase3 (#No.19677‐1‐AP, Proteintech), anti‐BAX (#5023, CST), anti‐Bcl‐2 (#4223, CST), anti‐ATM (#2873, CST), anti‐Phospho‐ATM Ser1981 (#5883, CST), anti‐BRD4 (#13440, CST), anti‐foxO3a (#12829, CST), anti‐C/EBPβ (#43095, CST), anti‐HMGB1 (#6893, CST), anti‐PARylation (#4335‐MC‐100, Trevigen), anti‐PARylation (#89190, CST), anti‐rabbit IgG (#7074, CST) and anti‐mouse IgG (#7076, CST).

Tempol (4‐Hydroxy‐2,2,6,6‐tetramethylpiperidine 1‐oxyl, #176141‐5G; 100 µM; 12 h), an antioxidant with no side effects or toxicity, was purchased from Sigma; CBP inhibitor (SGC‐CBP30; #S7256; 10^−5^ M; 24 h) was obtained from Selleckchem. ATM inhibitor (KU‐55933; #HY‐12016; 10 µM; 2 h); LDHA inhibitor (Galloflavin; #HY‐W040118; 10^−5^ M; 24 h) and CBP activator (CTB; #HY‐134964; 10^−4^ M; 24 h) were obtained from MedChemExpress. SIRT1 inhibitor (EX527; #A4181; 10^−5^ M; 24 h) and SIRT1 activator (SRT1720; #A4180; 5 µM; 24 h) were obtained from APExBio.

### 4.7 Plasmid, siRNA and recombinant adenoviruses

Myc‐PARP1, Myc‐PARP1(amino acids 1‐779), Myc‐PARP1(amino acids 203‐1014), Myc‐PARP1(amino acids 1‐476), Myc‐PARP1(amino acids 1‐203), Myc‐PARP1(amino acids 661‐1014), Myc‐PARP1(amino acids 484‐1014), Flag‐P300, Flag‐CBP, Flag‐PCAF, MYC‐GCN5, Flag‐SIRT1‐7, Myc‐LDHA were obtained from our previous studies. Mutations in PARP1 (Myc‐PARP1 5KR) were purchased from Shanghai GeneChem Company. After confirmation by DNA sequencing, the plasmids were transfected into cells using jetPrime Transfection reagent and the corresponding jet buffer (Polyplus PT‐114‐15). Cells were harvested after at least 48 h.

Specific siRNAs for LDHA and SIRT1 were purchased from RiboBio Co., Ltd. The three LDHA siRNAs are as follows: LDHA siRNA‐1: GGCAGCCTTTTCCTTAGAA. LDHA siRNA‐2: CTTGGAAGATAAGTGGTTT. LDHA siRNA‐3: GCTGGGAGTTCACCCATTA. The three SIRT1 siRNAs are as follows: SIRT1 siRNA‐1: GCCTGATGTTCCAGAGAGA. SIRT1 siRNA‐2: GACATGAACTATCCATCAA. SIRT1 siRNA‐3: GGATGAAAGTGAAATTGAA.

Recombinant adenoviruses for gene knockdown and overexpression were constructed for the infection of primary rat cardiomyocytes. For knockdown, short hairpin RNA sequences targeting rat PARP1 (GGAGACTTATGTATGATCCA), along with a nontargeting control (CCTAAGGTTAAGTCGCCCTCG), were synthesized, cloned and inserted into the BglII/SalI site of the pShuttle‐CMV shuttle vector (BR009, Hunan Fenghui Biotechnology) by Wuxi Genecefepharm Biotech. For overexpression, cDNA sequences encoding C‐terminal Myc‐tagged wild‐type PARP1 (Myc‐PARP1‐WT) or the mutant 5KR (Myc‐PARP1 mut 5KR) and C‐terminal Flag‐tagged CBP (Flag‐CBP) were inserted similarly, with an empty vector (Ad‐Vector) used as a control. Linearized shuttle vectors were co‐transformed with the adenoviral backbone plasmid pAdEasy‐1 into BJ5183‐AD‐1 electrocompetent Escherichia coli cells (Beijing Huayueyang Biotechnology) to generate full‐length adenoviral genomes via homologous recombination. The resulting recombinant plasmids were linearized with PacI (Thermo Fisher Scientific) and transfected into HEK‐293A (Cellverse) cells using Lipofectamine 3000 (Thermo Fisher Scientific) for virus production. Viruses were collected by repeated freeze‐thaw cycles and filtered through a  .45 µm filter, then amplified to final titres of Ad‐shNC at 2.3 × 10^9^ plaque‐forming units (PFU)/mL, Ad‐shPARP1 at 2.1 × 10^9^ PFU/mL, Ad‐Vector at 2.4 × 10^9^ PFU/mL, Ad‐Myc‐PARP1‐WT at 1.4 × 10^9^ PFU/mL and Ad‐Myc‐PARP1 mut 5KR at 1.2 × 10^9^ PFU/mL.

### 4.8 Mass spectrometry

The cells overexpressing Myc‐PARP1 were harvested after transfection for 48 h. Cells were lysed and immunoprecipitated with anti‐Myc antibodies according to the co‐immunoprecipitation protocol described previously. The immunoprecipitated proteins were then subjected to western blotting. The bands corresponding to PARP1 were excised from the gel, digested with trypsin, and subjected to liquid chromatography‐tandem mass spectrometry (LC‐MS/MS) analysis. MS experiments were performed on a nanoscale UHPLC system (EASY‐Nlc1000 from Proxeon Biosystems) connected to an Orbitrap Q‐Exactive equipped with a nanoelectrospray source (Thermo Fisher Scientific). Raw data were processed using Proteome Discoverer (PD, version 2.1), and MS/MS spectra were searched against the reviewed Swiss–Prot human proteome database. Only peptides with at least six amino acids in length were considered. Identification of peptides and proteins was screened by PD to control the false discovery rate (FDR) to less than 1%. Protein identification requires at least one unique peptide.

### 4.9 Co‐immunoprecipitation (Co‐IP) and Western blot analysis

Cells were lysed for 30 min on ice with IP lysis buffer (50 mM Tris‐HCl at PH 7.4, 1%Triton X‐100, 1% NP40, 150 mM NaCl, 1 mM EDTA, 0.25% sodium deoxycholate) supplemented with protease inhibitor cocktails, and with phosphorylation protease inhibitor when necessary. Proteins were collected after centrifuging the lysates at 13 500 rpm for 20 min at 4°C. Then, the protein concentration was assessed by G250 and 40 µg of cell lysate were used for the preparation of samples. For Co‐IP, cells were lysed using an IP lysis buffer similar to the western blot analysis method. Afterwards, primary antibodies were added to the cell lysates and mixed by rotation for 1 h, and then protein A/G beads (Santa Cruz) were added to the mixture. The mixture was incubated overnight at 4°C. After Co‐IP, the samples were washed with PBS at 4°C three times. Protein samples were eluted with 2×loading. Protein samples or eluates were subjected to 6%, 8%, 10%, 12% or 15% SDS‐PAGE and then transferred to PVDF membrane (Millipore, IPVH00010) for 30 to 180 min, depending on the target molecular weight range. The membranes were probed with specific primary antibodies at 4°C overnight after blocking in 5% BSA in TBST for one hour at room temperature. The membranes were then washed with TBST three times, followed by incubation with HRP‐conjugated secondary antibody at room temperature for two hours. After three washes, bands were detected by using the enhanced chemiluminescence detection kit (Thermo Fisher Scientific, 32106) and visualized via the DNR western blot detection system.

### 4.10 Lactate measurements

The intracellular lactate concentrations were measured using the CheKine Lactate Assay Kit (KTB1100, Abbkine) in accordance with the manufacturer's instructions: cell counting was performed first, and then the cells were collected in a centrifuge tube, washed with cold PBS, centrifuged, and the supernatant discarded. Cells were broken by sonication with Lactate Assay Buffer at a ratio of 1 mL/5 million for 5 min in an ice bath, then centrifuged at 12 000*g* for 5 min at 4°C, and the supernatant was placed on ice for measurement. For the measurement of serum lactic acid, the mouse serum was diluted five times and placed on ice. The zymograph was preheated for 30 min, and the wavelength was adjusted to 450 nm. 50 µL of the sample to be tested was mixed with 50 µL of Working Reagent and incubated for 30 min at 37°C, protected from light. The absorbance of the Lactate Assay Buffer and different concentrations of Standard at 450 nm were used to calculate the standard curve. The absorbance of the sample at 450 nm was measured directly, and the lactic acid content was calculated from the standard curve.

### 4.11 in vitro PARP1 lactylation and delactylation

To determine the direct effects of CBP on PARP1 lactylation, Myc‐PARP1 and Flag‐CBP plasmids were transfected into HL‐1 cells for 48 h, followed by purification with anti‐Myc beads or anti‐Flag beads, respectively. These purified proteins were added to the reaction buffer containing 50 mM 4‐(2‐hydroxyethyl)‐1‐piperazineethanesulfonic acid (HEPES), pH 7.8, 30 mM KCl, 0.25 mM EDTA, 5.0 mM MgCl_2_, 5.0 mM sodium butyrate, 2.5 mM dithiothreitol (DTT) and lactyl‐CoA (provided by Dr Yingming Zhao). After incubation at 37°C for 20 min, reaction products were denatured with 2×LDS loading buffer at 90°C for 10 min for western blotting.

To determine the direct effects of SIRT1 on PARP1 delactylation, Myc‐PARP1, Flag‐CBP and Flag‐SIRT1 plasmids were transfected into HL‐1 cells for 48 h, followed by purification with anti‐Myc beads or anti‐Flag beads, respectively. These purified proteins were added to the reaction buffer. After incubation at 37°C for 20 min, reaction products were denatured with 2×LDS loading buffer at 90°C for 10 min for western blotting.

### 4.12 Immunohistochemistry

Formalin‐fixed heart tissues were embedded in paraffin and cut into 4 µm sections. The sections were dewaxed in xylene, rehydrated in a series of descending percentages of ethanol, and boiled in Tris‐EDTA solution for antigen retrieval. The UltraSensitive SP IHC kit (MXB) was used for immunostaining. After blocking, the slides were incubated with primary antibody for PARylation (#89190, Cell Signaling Technology) at 37°C. After washing, the sections were immersed with biotin‐conjugated immunoglobulin G secondary antibody from the immunohistochemistry kit for 20 min, stained by DAB (MXB) for 2 min, and then counter‐stained in hematoxylin for 2 min.

### 4.13 Flow cytometric analysis

To measure apoptosis, cells were treated with doxorubicin for at least 12 h and then incubated with Annexin V‐FITC Apoptosis Detection Kit (Dojindo Laboratories) according to the manufacturer's instructions. Briefly, 2 × 10^5^ cells and supernatant were collected by using non‐EDTA trypsin. After washing with PBS, the cell pellets were resuspended in binding buffer. Cells in each group were added with 5 µL Annexin V and 5 µL PI, and then incubated for 30 min away from light. APC and PI channels were used to detect apoptosis. Flow cytometry was performed using the LSRFortessa (BD), and flow data were analysed using FlowJo Software v.10 (Tree Star). The apoptotic cells were in the Q2+Q4 regions.

### 4.14 In vitro cell proliferation and viability analysis

The proliferation and viability of rat primary cardiomyocytes were measured by a Microplate Reader Bio‐Rad microplate reader (Model 680; Bio Rad Laboratories, Inc.) at 450 nm using the Cell Counting Kit‐8 (CCK‐8, Dojindo), following the manufacturer's instructions.

PrestoBlue assay (Thermo Fisher Scientific) was performed according to the manufacturer's instructions. PrestoBlue reagent was added in a 1:10 ratio to cancer cells (MV4‐11, SKOV3, MDA‐231 and Raji) and incubated for 30 min at 37°C. The intensity of fluorescence was measured using the Synergy HTX plate‐reader (Biotek) or ThermoVarioskan LUX Multi‐mode Microplate Reader (Thermofisher).

### 4.15 Detection of SIRT1 and CBP enzyme activity

SIRT1 activity was measured using the Sirtuin Fluorometric Assay Kit (CycLex, Ina). Total protein extracted from mice myocardial tissues was immunoprecipitated with rabbit anti‐SIRT1 polyclonal antibody and protein G agarose beads. After overnight incubation at 4°C, the pellet was transferred to a new tube. Subsequently, 15 µL of the precipitate and 35 µL of the reaction mixture (50 mmol/L Tris‐HCl, 0.25 m AU/mL lysozyme, 1 µM trichostatin A, 800 µM NAD^+^ and 20 µM fluorinated substrates) were mixed to react. The fluorescence intensity (excitation: 490 ± 10 nm, emission: 530 ± 15 nm) was detected using a microplate fluorometer with the protein concentration as standard.

CBP HAT activity was measured using the HAT fluorometric assay kit (Active Motif). Nuclear protein extracted from mice myocardial tissues was immunoprecipitated with rabbit anti‐CBP polyclonal antibody and protein G agarose beads. After overnight incubation at 4°C, the precipitate pellet was collected and transferred to a new tube. Subsequently, 15 µL of immunoprecipitate was mixed with 35 µL reaction buffer mixture (50 mM Tris‐HCl, 1 mM DTT, 10 µM trichostatin A, 100 µM acetyl‐CoA and fluorogenic histone H3 peptide substrate) to initiate the enzymatic reaction. The resulting fluorescence intensity (excitation: 380 ± 10 nm, emission: 460 ± 15 nm) was recorded via a microplate fluorometer with the protein concentration as standard.

### 4.16 Quantitative real‑time PCR

Cells were lysed in Trizol Reagent (Thermo Fisher Scientific) and extracted as per the manufacturer's instructions. Reverse transcription was performed using the PrimeScript RT Master Mix (Takara). Quantitative RT‐PCR of target genes was performed with GoTaq qPCR Master Mix (Promega) or TB Green Premix Ex Taq II kit (Takara) using QuantStudio 12K Flex (Thermo Fisher Scientific). Gene expression was calculated relative to GAPDH and normalized to the control. Each biological replicate is the average of technical duplicates. The primers designed from Sangon Biotech Co., Ltd are as follows: MYC (forward primer: 5′‐GGCTCCTGGCAAAAGGTCA‐3′; reverse primer: 5′‐CTGCGTAGTTGTGCTGATGT‐3′), CCNB1 (forward primer:5′‐TGTTGGTTTCTGCTGGGTGT‐3′; reverse primer:5′‐TGCCATGTTGATCTTCGCCT‐3′), CDK1 (forward primer:5′‐GGAAACCAGGAAGCCTAGCATC‐3′; reverse primer: 5′‐GGATGATTCAGTGCCATTTTGCC‐3′), BIRC5 (forward primer:5′‐AGGACCACCGCATCTCTACAT‐3′; reverse primer: 5′‐AAGTCTGGCTCGTTCTCAGTG‐3′), GAPDH (forward primer: 5′‐TGTGAGGAGGGGAGATTCAG‐3′; reverse primer: 5′‐CCTCCAAGGAGTAAGACCCC‐3′).

### 4.17 Statistical analysis

Data are presented as the mean ± standard deviation (SD). For the in vitro assay, the number of cells was sufficiently large (hundreds of thousands of cells) that the ratio (detected mean indicator) calculated is the amount of protein expression divided by the internal reference, and according to the central limit theorem, the detected mean indicator approximately obeys a normal distribution, and the mean indicators of three independent repeated experiments obey a normal distribution. The levels of intracellular lactate, oxidative stress, apoptosis, cell proliferation and cellular total PARylation represent the global cellular status of the overall cell, and based on the central limit theorem, the detected mean level approximately obeys a normal distribution, and we assume data from in vitro experiments are distributed normally. For the analysis of in vivo experiments (the sample sizes all equal to 10), the normal distribution was tested by the Shapiro–Wilk test and homogeneity of variance was tested by the F test (two‐group comparison) or Bartlett's test (more than two group comparisons) with a threshold *p*‐value of  .05. For normally distributed data with equal variance, the unpaired *t*‐test (two‐tailed Student *t*‐test) was used for 2‐group studies, one‐way ANOVA with Tukey multiple comparisons test was utilized for comparing the effect of treatment for more than 2 groups and two‐way ANOVA with Bonferroni multiple comparisons test was used when two conditions were considered between the groups. For data that failed normality testing or equal variance testing, the Mann–Whitney test was used to compare 2‐group data, and the Kruskal–Wallis test, followed by the Dunn multiple comparison test, was performed for multiple group comparison. Bonferroni corrections were applied to multiple comparisons to adjust for the inflation of the type I error, and *p*‐values were adjusted for multiple comparisons when applicable. Detailed methods for assessing significance are included in each figure legend, as well as the number of biological replicates in each experiment group. SPSS version 22.0 software (SPSS Inc.) and GraphPad Prism 9.0 Software (GraphPad) were used for all statistical analyses, with *p *< .05 indicating statistical significance. A representative image reflecting the average result of each experiment was selected for the relevant figures.

## AUTHOR CONTRIBUTIONS

Yingxian Sun came up with the research idea, study design, and concept. Wenbin Wang supervised the study, performed experiments, acquired data, analyzed data and wrote the manuscript. Yingxian Sun critically reviewed it and approved the final manuscript.

## CONFLICT OF INTEREST STATEMENT

The authors declare no conflict of interest.

## Supporting information




SUPPORTING INFORMATION

## Data Availability

The data generated in the analysis process is available from the corresponding author upon reasonable request.
