# Supplementary material for: ROS‐ATM‐CBP axis‐mediated PARP1 lactylation aggravates doxorubicin‐induced cardiotoxicity
Source: Clin Transl Med. 2026 Aug 17;16(8):e70745. doi: 10.1002/ctm2.70745 (PMC13478851; doi:10.1002/ctm2.70745)
Supplement: Supplementary file 1 [file CTM2-16-e70745-s001.docx]

**Figure S1. CBP is the lactyltransferase for PARP1 lactylation
A and B,** CBP can catalyze lactylation of PARP1. **C and D,** Quantification for endogenous PARP1 lactylation in Figure 1F and G. **E,** Quantification for Full-length Myc-PARP1 or six truncated Myc-PARP1 lactylation in Figure 1H. **F,** Full-length Myc-PARP1 or truncated Myc-PARP1 plasmids (1-1014, 1-476, 1-203, 484-1014) were transfected respectively with or without co-overexpression of Flag-CBP, and total lysates were subjected to immunoprecipitation with anti-Myc antibody, followed by detection of Pan-KLA. **G,** Full-length Myc-PARP1 or truncated Myc-PARP1 plasmids (1-1014, 1-476, 1-203, 484-1014) were transfected respectively with or without doxorubicin treatment, and total lysates were subjected to immunoprecipitation with anti-Myc antibody, followed by detection of Pan-KLA. **H,** Quantification for Myc-PARP1-WT or Myc-PARP1-mut 5KR lactylation in Figure 1J. **I,** Quantification for Myc-PARP1-WT or Myc-PARP1-mut 5KR lactylation in Figure 1K. Based on the central limit theorem, the data were considered to be normally distributed. Relative protein levels were calculated as fold changes vs the first group. Data are presented as mean ± SD. **B,** Statistical significance was assessed by 1-way ANOVA with Tukey multiple comparisons test (*P* values adjusted for 10 comparisons). **C and D,** the unpaired t test (2-tailed Student t test) was used to compare 2 groups. **E,** Statistical significance was assessed by 1-way ANOVA with Tukey multiple comparisons test (*P* values adjusted for 21 comparisons). **F and G,** Statistical significance was assessed by 1-way ANOVA with Tukey multiple comparisons test (*P* values adjusted for 28 comparisons). **H and I,** Statistical significance was assessed by 2-way ANOVA with Bonferroni multiple comparisons test (*P* values adjusted for 6 comparisons). ns, *P*>0.05; **P*<0.05; ***P*<0.01; ****P*<0.001. All immunoblots had three biological repetitions, with similar results.

**Figure S2. SIRT1 is the delactylase for PARP1
A and B,** SIRT1 can specifically erase lactylation of PARP1. **C-E,** Quantification for endogenous PARP1 lactylation in Figure 2D-F. **F,** Quantification for Myc-PARP1-WT or Myc-PARP1-mut 5KR lactylation in Figure 2G. **G,** Quantification for Myc-PARP1-WT or Myc-PARP1-mut 5KR lactylation in Figure 2H. Based on the central limit theorem, the data were considered to be normally distributed. Relative protein levels were calculated as fold changes vs the first group. Data are presented as mean ± SD. **B,** Statistical significance was assessed by 1-way ANOVA with Tukey multiple comparisons test (*P* values adjusted for 28 comparisons). **C-E,** the unpaired t test (2-tailed Student t test) was used to compare 2 groups. **F and G,** Statistical significance was assessed by 2-way ANOVA with Bonferroni multiple comparisons test (*P* values adjusted for 6 comparisons). ns, *P*>0.05; **P*<0.05; ***P*<0.01; ****P*<0.001. All immunoblots had three biological repetitions, with similar results.

**Figure S3. LDHA regulates lactate production and PARP1 lactylation
A-D,** Quantification for endogenous PARP1 lactylation in Figure 3E-H. **E,** Quantification for Myc-PARP1-WT or Myc-PARP1-mut 5KR lactylation in Figure 3I. **F,** Quantification for Myc-PARP1-WT or Myc-PARP1-mut 5KR lactylation in Figure 3J. Based on the central limit theorem, the data were considered to be normally distributed. Relative protein levels were calculated as fold changes vs the first group. Data are presented as mean ± SD. **A-D,** the unpaired t test (2-tailed Student t test) was used to compare 2 groups. **E and F,** Statistical significance was assessed by 2-way ANOVA with Bonferroni multiple comparisons test (*P* values adjusted for 6 comparisons). ns, *P*>0.05; **P*<0.05; ***P*<0.01; ****P*<0.001. All immunoblots had three biological repetitions, with similar results.

**Figure S4. ROS-ATM-CBP signaling pathway activates PARP1 lactylation
A,** Representative immunoprecipitation, western blots and quantification for detecting PARP1 lactylation. HL-1 cells were stimulated by H_2_O_2_ (150μM for 0, 30, 60 minutes). **B,** Representative immunoprecipitation, western blots and quantification for detecting PARP1 lactylation. HL-1 cells were pre-treated with or without Tempol (100μM, 12h). Cells were lysed after treatment with or without doxorubicin (1μM for 4 hours). **C-F,** Representative immunoprecipitation, western blots and quantification for detecting the interaction of PARP1 with CBP/SIRT1 in mice. Doxorubicin was intraperitoneally injected once a week with a dose of 5 mg/kg or the same volume of saline for 4 weeks (20 mg/kg cumulative dose of DOX). **G-H,** Representative Western blots and quantification of PARP1 lactylation in rat primary cardiomyocytes under doxorubicin (1μM for 24h) treatment. **I,** Representative immunoprecipitation, western blots and quantification for detecting PARP1 lactylation. HL-1 cells were pre-treated with or without ATM inhibitor (10μM; 2h). Cells were lysed after treatment with or without doxorubicin (1μM for 24h). **J,** Representative immunoprecipitation, western blots and quantification for detecting the interaction of PARP1 with CBP. HL-1 cells were pre-treated with or without ATM inhibitor (10μM; 2h). Cells were lysed after treatment with or without doxorubicin (1μM for 24h). **A,** Statistical significance was assessed by 1-way ANOVA with Tukey multiple comparisons test (*P* values adjusted for 3 comparisons). **B,** Statistical significance was assessed by 2-way ANOVA with Bonferroni multiple comparisons test (*P* values adjusted for 3 comparisons). **C-F,** the unpaired t test (2-tailed Student t test) was used to compare 2 groups. **G-J,** Statistical significance was assessed by 2-way ANOVA with Bonferroni multiple comparisons test (*P* values adjusted for 6 comparisons). Data are presented as mean ± SD. **P*<0.05; ***P*<0.01; ****P*<0.001. All immunoblots had three biological repetitions, with similar results.

**Figure S5.** **CBP exacerbates DOX‑induced myocardial injury primarily through the PARP1 lactylation-PARylation axis
A,** Experimental design for Figure 5. Co-infection with Ad-shPARP1 and either Ad-Myc-PARP1-WT or -mut 5KR with or without Ad-Flag-CBP, followed by treatment with or without 1μM DOX for 24h. **B and G,** Representative immunoprecipitation, western blots and quantification for detecting BRD4, FOXO3α, C/EBPBβ, and HMGB1 PARylation in HL-1 cells with or without DOX (1μM for 24h) treatment. **C-F and H,** Representative immunoprecipitation, western blots and quantification for the interaction between Myc-PARP1-WT or Myc-PARP1-mut 5KR with BRD4, FOXO3α, C/EBPBβ, and HMGB1 in HL-1 cells with or without DOX (1μM for 24h) treatment. **G, H,** Statistical significance was assessed by 2-way ANOVA with Bonferroni multiple comparisons test (*P* values adjusted for 6 comparisons). Data are presented as mean ± SD. ***P*<0.01; ****P*<0.001. All immunoblots had three biological repetitions, with similar results.

**Figure S6. CBP inhibition enhances the antitumor efficacy of doxorubicin
A-D,** Normalized viability in acute leukemia MV4‑11 **(A)**, ovarian cancer SKOV3 **(B)**, triple‑negative breast cancer MDA‑231 **(C)**, and Burkitt’s lymphoma Raji **(D)** were measured using the PrestoBlue assay (n=6 per group). **E-H,** qPCR analysis of MYC, CCNB1, CDK1 and BIRC5, normalized to glyceraldehyde 3-phosphate dehydrogenase and control (n=6 per group). Statistical significance was assessed by 1-way ANOVA with Tukey multiple comparisons test (*P* values adjusted for 3 comparisons). Data are presented as mean ± SD. ***P*<0.01; ****P*<0.001.
